# Human ignitions on private lands drive USFS cross-boundary wildfire transmission and community impacts in the western US

**DOI:** 10.1038/s41598-022-06002-3

**Published:** 2022-02-15

**Authors:** William M. Downing, Christopher J. Dunn, Matthew P. Thompson, Michael D. Caggiano, Karen C. Short

**Affiliations:** 1 Department of Forest Engineering, Resources and Management, College of Forestry, Oregon State University, Corvallis, OR 97331 USA; 2 Human Dimensions Program, USDA Forest Service Rocky Mountain Research Station, Ft. Collins, CO 80526 USA; 3 Department of Forest & Rangeland Stewardship, Colorado State University, Fort Collins, CO 80523 USA; 4 Fire, Fuel, and Smoke Science Program, USDA Forest Service Rocky Mountain Research Station, Missoula, MT 59808 USA

**Keywords:** Fire ecology, Natural hazards

## Abstract

Wildfires in the western United States (US) are increasingly expensive, destructive, and deadly. Reducing wildfire losses is particularly challenging when fires frequently start on one land tenure and damage natural or developed assets on other ownerships. Managing wildfire risk in multijurisdictional landscapes has recently become a centerpiece of wildfire strategic planning, legislation, and risk research. However, important empirical knowledge gaps remain regarding cross-boundary fire activity in the western US. Here, we use lands administered by the US Forest Service as a study system to assess the causes, ignition locations, structure loss, and social and biophysical factors associated with cross-boundary fire activity over the past three decades. Results show that cross-boundary fires were primarily caused by humans on private lands. Cross-boundary ignitions, area burned, and structure losses were concentrated in California. Public lands managed by the US Forest Service were not the primary source of fires that destroyed the most structures. Cross-boundary fire activity peaked in moderately populated landscapes with dense road and jurisdictional boundary networks. Fire transmission is increasing, and evidence suggests it will continue to do so in the future. Effective cross-boundary fire risk management will require cross-scale risk co-governance. Focusing on minimizing damages to high-value assets may be more effective than excluding fire from multijurisdictional landscapes.

## Introduction

Global fire dynamics are shifting dramatically in the twenty-first century. Changing fire regimes are intersecting with the consequences of historical fire and forest management practices^1–3^, as well as increasing expansion of the wildland urban interface (WUI)^4^. Rising temperatures, increased drought, longer fire seasons, and earlier snowmelt have all been associated with more burning in recent decades^5–7^. In addition, the accumulation of wildland fuels resulting from fire suppression and other land management practices is further increasing fire activity. Prior to Anglo-European colonization in the western United States, fire burned with a wide range of extents, frequencies, and severities, limited by the availability of fuel, favorable fire weather, and ignition sources^8^. As European colonization intensified, historical fire regimes were significantly altered by factors including the cessation of indigenous burning practices and the widespread adoption of aggressive fire suppression^9–11^. Meanwhile, human development in and around wildlands expanded by 41% between 1990 and 2010, making the WUI the fastest growing land use type in the US^4^. Increased development has resulted in both more risk and more loss. Millions of homes in the WUI are threatened by wildfires each year^12^, and the annual number of structures lost to wildfire increased by 300% between 1990 and 2014^13^.

As the WUI expands, there is often increased socio-ecological conflict, whereby anthropogenic pressures have negative impacts on natural resources; and natural disturbances, such as fire, have negative consequences for human communities^14,15^. The dramatic expansion of the WUI has exacerbated the wildfire problem by resulting in more human-caused ignitions^16^, which are now the dominant cause of fire in the US^17^ and the primary source of fire risk to communities^12^. Each year in the western United States (US) federal agencies undertake increasingly costly (~ $5 billion year^−1^) efforts to suppress wildfires and reduce social, economic, and ecological wildfire damages^18^. However, increased fire suppression has not translated into decreased damages. Wildfires are getting bigger, more destructive, and more deadly^19–21^. In California alone, the wildfires of 2018 burned 7,400 km^2^ and resulted in the deaths of 103 people, the loss of 22,000 structures, and estimated economic damages totaling $148.5 billion^20,22^.

The tension between ecological processes (e.g., fire) and social processes (e.g., WUI development) in mixed ownership landscapes is brought into stark relief when fire ignites on one land tenure and spreads to other ownerships, especially when it results in severe damages to communities on private lands and/or highly valued natural resources on publicly managed wildlands. These cross-boundary (CB) wildfires present particularly acute management challenges because the responsibilities for preventing ignitions, stopping fire spread, and reducing the vulnerability of at-risk, high-value assets are often dispersed among disparate public and private actors with different objectives, values, capacity, and risk tolerances^23–25^. Some CB risk mitigation strategies exist, such as fire protection exchanges, which transfer suppression responsibility from one agency (e.g., state) to another (e.g., U.S. Forest Service), and CB fuel treatment agreements, which allow managers to influence components of wildfire risk beyond their jurisdictional boundaries^2,26^. Improving CB wildfire risk management has been identified as a top national priority^27^, but effective, landscape-scale solutions are not readily apparent.

A common narrative used to describe CB fire is as follows: a wildfire ignites on remote public lands (e.g., US Forest Service), spreads to a community, showers homes with embers, and results in structure loss and fatalities^23,25,28^. In this framing, public land management agencies bear the primary responsibility for managing and mitigating CB fire risk, with effort focused on prevention, hazardous fuel reduction, and suppression—largely reinforcing the dominant management paradigm of fire exclusion^29,30^. An alternative risk management framing of this challenge has emerged, starting with the axiom that CB fire transmission is inevitable in fire-prone mixed ownership landscapes and that private landowners and homeowners are the actors best positioned to reduce fire risk to homes and other high-value assets regardless of where the fire starts^31^. In the absence of a broad-scale empirical assessment of CB fire transmission, it is difficult to determine which of these narratives more accurately reflects the nature of the problem, and whether CB fire risk management is best framed in terms of reducing fire transmission from public lands or decreasing the exposure and vulnerability of high-value developed assets on private lands.

Despite advances in simulated wildfire hazard assessments and legislation and policy promoting CB wildfire risk engagement, important knowledge gaps remain regarding the causes, ignition locations, structure loss, and social and biophysical factors associated with recent CB fire activity. One possibility is that all else being equal, CB fire activity simply increases proportionally with the number and extent of jurisdictional boundaries available for fires to cross. Alternatively, CB burning may be primarily controlled by the degree to which a landscape’s temperature, precipitation, and fuels promote ignition and fire spread^32,33^. If biophysical drivers were dominant, we would expect that CB area burned would essentially mirror area burned by fire that did not cross jurisdictional boundaries, and we might anticipate that more CB fire would occur in areas where fire intensities often exceed the capacity of firefighters to prevent fire spread. A third possibility is that social factors such as population density and road networks may override climatic and fire behavior factors, as has been observed in a number of fire-prone regions^16,34,35^. These uncertainties make it difficult to prioritize specific mitigation actions and identify the actors best positioned to manage different aspects of fire transmission risk. Understanding why there is more CB fire activity in some places and less in others could help target mitigations based on causal factors, but the social and biophysical factors associated with CB fire transmission have not been systematically explored across the western US.

In this paper we present an empirical assessment of recent CB fire activity in the western US. We use the United States Department of Agriculture Forest Service (USFS) National Forest System and surrounding ownerships as our focal domain, and define CB fires as those fires that burned both USFS lands and other land tenures. The USFS is the largest fire management organization in the US and administers approximately 75% of federal wildfire appropriations^18^. We began by leveraging comprehensive fire occurrence, area burned, and structure loss datasets to undertake a spatially explicit, retrospective analysis of fire transmission across USFS jurisdictional boundaries. Next, we analyzed these spatial data using a machine learning statistical modeling approach to evaluate the strength and shape of relationships between CB fire activity and suite of biophysical and social factors. Specifically, we asked: (1) How much CB fire has occurred, and how have fire transmission rates changed in the last three decades? (2) Where, and on what ownerships, is CB activity most common? (3) Do the most destructive wildfires originate primarily on public lands managed by the USFS and spread to communities? (4) What are the social and biophysical factors most strongly related to variability in CB area burned and CB ignition densities on USFS and private lands, the two dominant sources and recipients of CB in our study domain?

## Results

**Figure 1:**
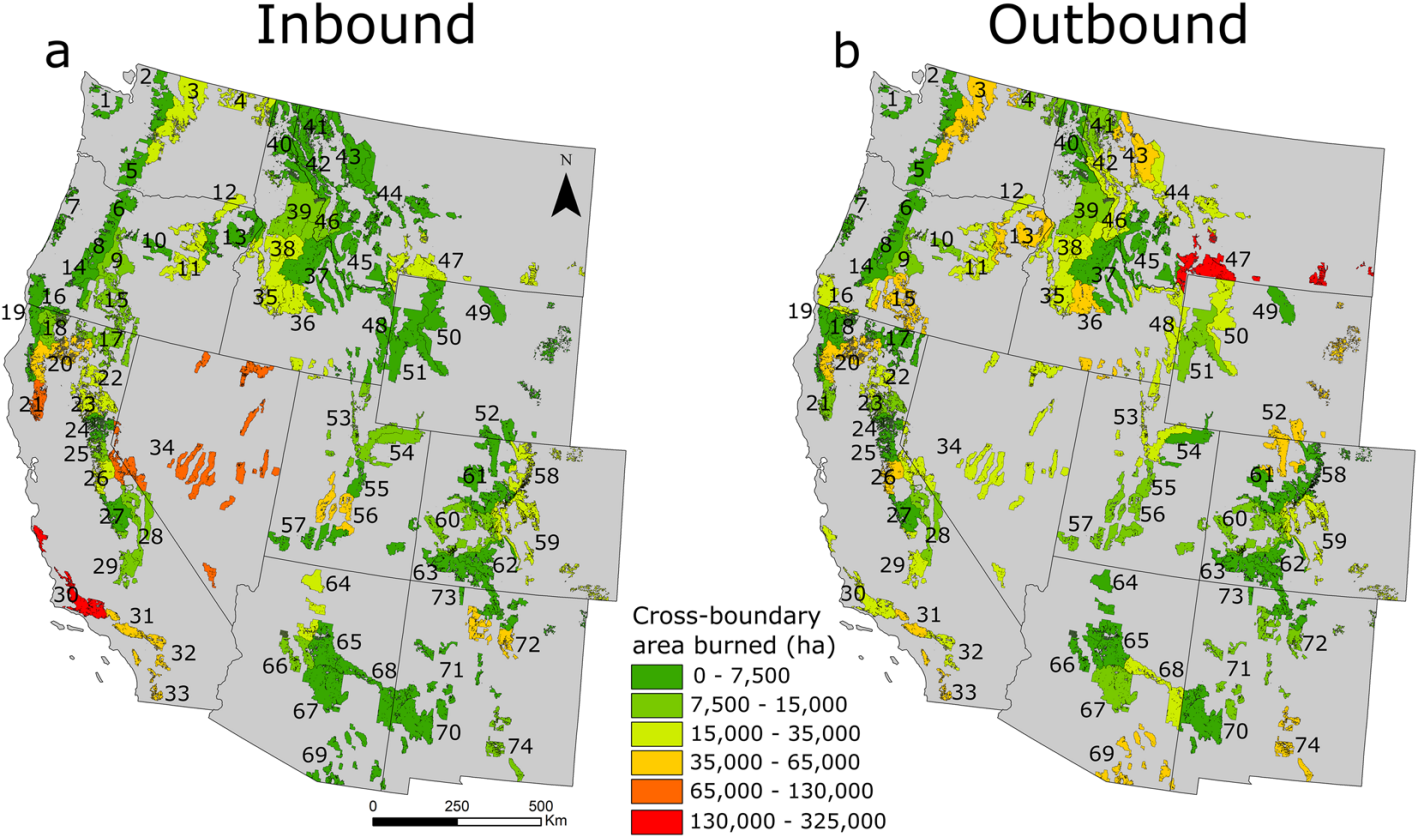
Area burned by CB fires that impacted USFS lands. Polygons represent USFS national forests. (**a**) USFS area burned by fires ignited on non-USFS lands (inbound). (**b**) Area burned outside of national forests by fires ignited on USFS lands (outbound). 1. Olympic, 2. Mt. Baker-Snoqualmie, 3. Okanogan-Wenatchee, 4. Colville, 5. Gifford Pinchot, 6. Mt. Hood, 7. Siuslaw, 8. Willamette, 9. Deschutes, 10. Ochoco, 11. Malheur, 12. Umatilla, 13. Wallowa-Whitman, 14. Umpqua, 15. Fremont-Winema, 16. Rogue River-Siskiyou, 17. Modoc, 18. Klamath, 19. Six Rivers, 20. Shasta-Trinity, 21. Mendocino, 22. Lassen, 23. Plumas, 24. Tahoe, 25. Eldorado, 26. Stanislaus, 27. Sierra, 28. Inyo, 29. Sequoia, 30. Los Padres, 31. Angeles, 32. San Bernardino, 33. Cleveland, 34. Humboldt-Toiyabe, 35. Boise, 36. Sawtooth, 37. Salmon-Challis, 38. Payette, 39. Nez Perce-Clearwater, 40. Idaho Panhandle, 41. Kootenai, 42. Lolo, 43. Flathead, 44. Helena-Lewis and Clark, 45. Beaverhead-Deerlodge, 46. Bitterroot, 47. Custer-Gallatin, 48. Caribou-Targhee, 48. Bighorn, 50. Shoshone, 51. Bridger-Teton, 52. Medicine Bow-Routt, 53. Uinta-Wasatch-Cache, 54. Ashley, 55. Manti-La Sal, 56. Fishlake, 57. Dixie, 58. Arapaho-Roosevelt, 59. Pike-San Isabel, 60. Grand Mesa Uncompahgre-Gunnison, 61. White River, 62. Rio Grande, 63. San Juan, 64. Kaibab, 65. Coconino, 66. Prescott, 67. Tonto, 68. Apache-Sitgreaves, 69. Coronado, 71. Cibola, 72. Santa Fe, 73. Carson, 74. Lincoln.

**Figure 2:**
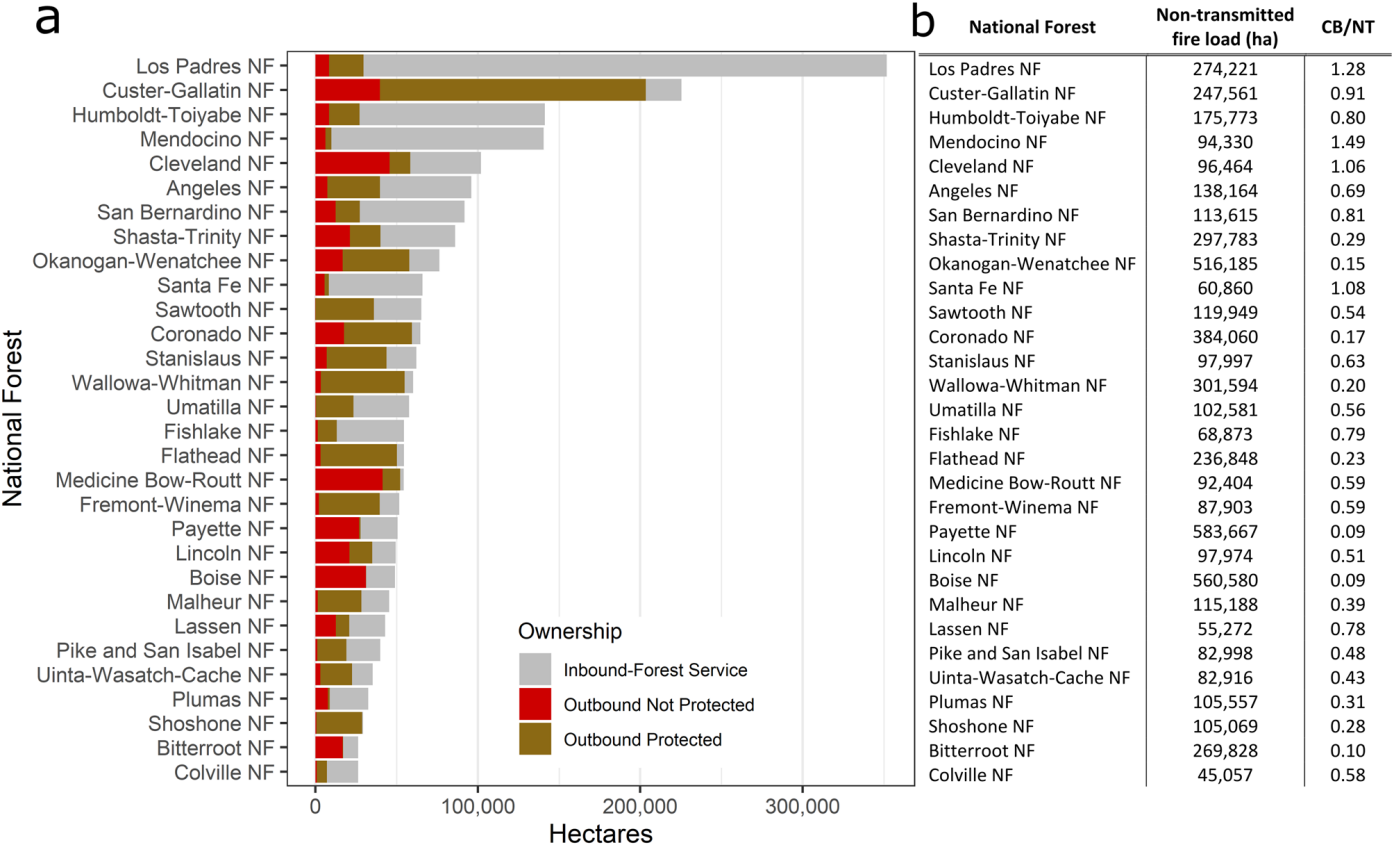
(**a**) National forests ranked according to area burned by CB fire between 1992 and 2019. Some forests are net receivers of inbound fire (e.g., Los Padres), while others are net transmitters (e.g., Custer-Gallatin). (**b**) Total non-transmitted fire load and the ratio of CB area burned (inbound and outbound) to the area burned by non-transmitted fire (CB/NT). CB fire is a major contributor to area burned in and around some national forests (e.g., Mendicino) and not others (e.g., Payette).

**Figure 3:**
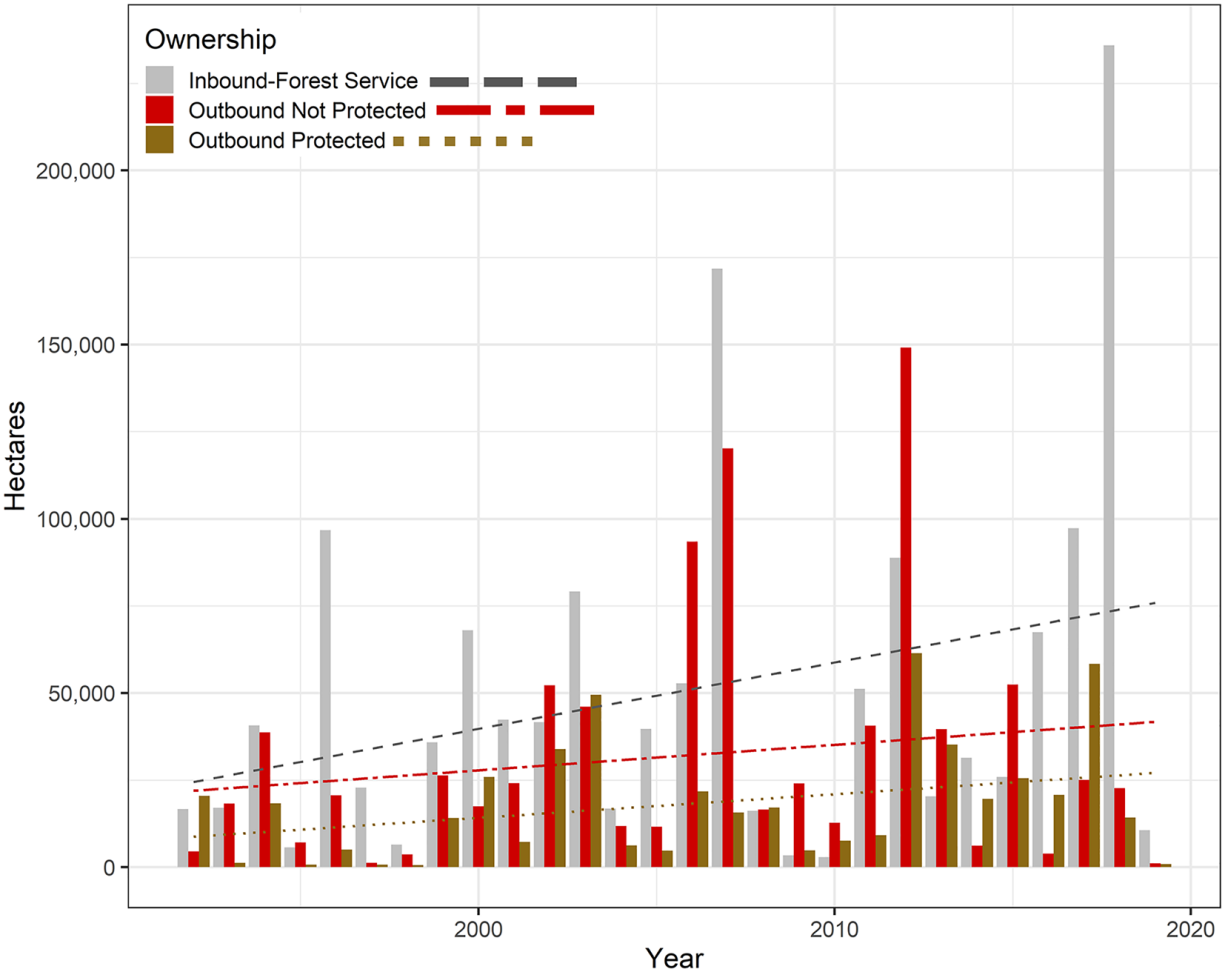
Area burned by CB fires derived from FIRESTAT data and binned by ownership category. Blue dots represent decadal averages of inbound and outbound acres combined. CB fire activity increased substantially during our study period. Area burned on USFS lands by fires originating on other ownerships (“inbound”, gray) has increased more rapidly than area burned on non-USFS lands. Ownership categories are described in more detail in the Methods.

A total of 6.9 million ha burned in CB fires between 1992 and 2019, approximately half on USFS lands (3.5 million ha) and half on other ownerships (3.4 million ha). CB area burned varied by five orders of magnitude (8 ha—351,625 ha) among the 74 national forests surveyed. Fire transmission was concentrated in a relatively small group of national forests located primarily in California (Figs. 1 and 2). We observed substantial variation in the relative amounts of area burned by fires ignited off USFS lands that spread to national forests (“inbound”) and area burned by fires that ignited on USFS lands and spread to other ownerships (“outbound”). CB area burned exhibited substantial inter-annual variability along with clear evidence of a general increase over the last three decades (Fig. 3). Inbound area burned on USFS lands increased at a higher rate (1,905 ha year^−1^) than outbound area burned on lands the USFS has suppression responsibility according to protection exchanges (678 ha year^−1^) and lands not protected by the USFS (732 ha year^−1^).

**Figure 4:**
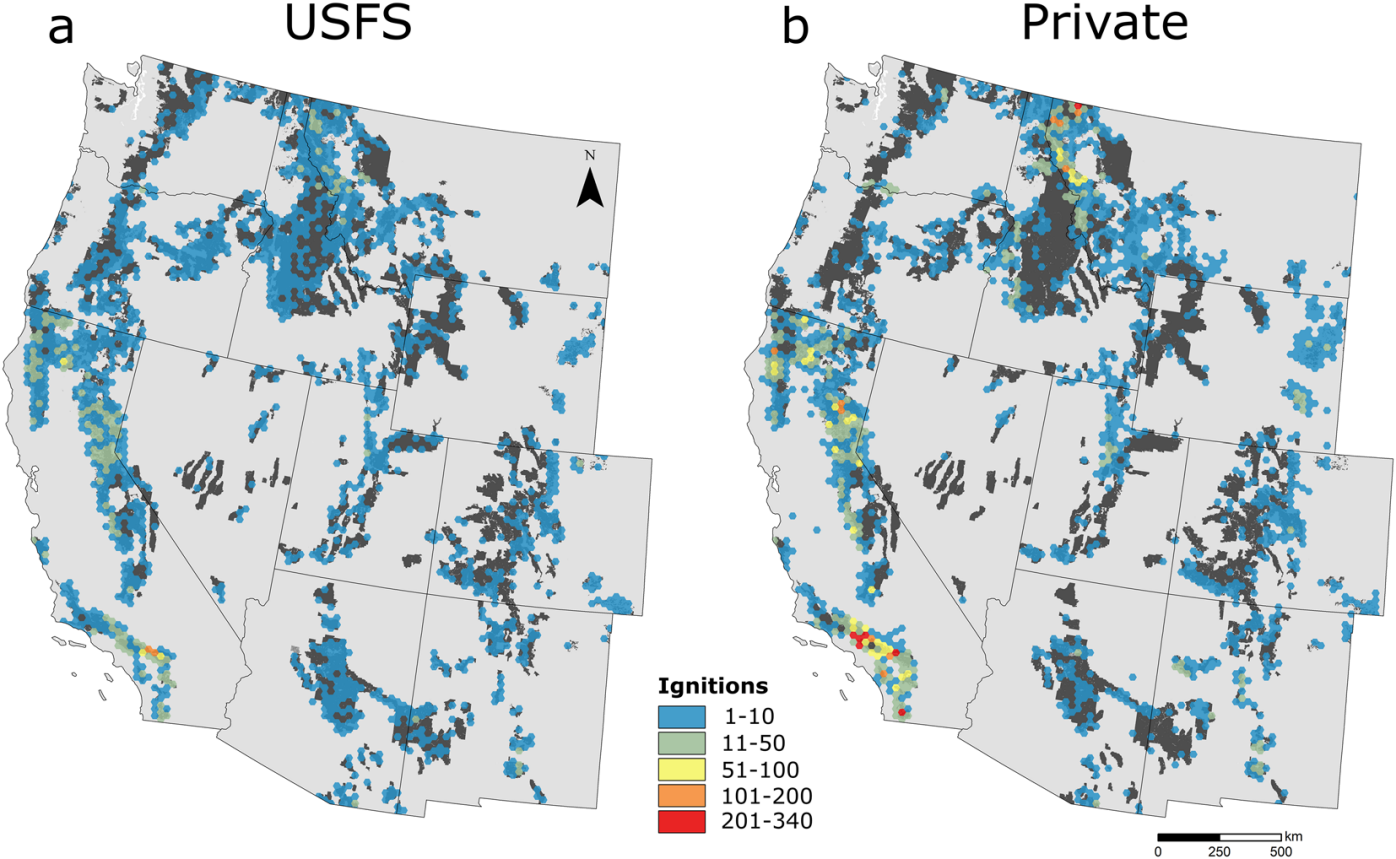
CB ignition densities derived from FPA FOD and FIRESTAT databases for fires originating on (**a**) USFS and (**b**) private lands between 1992 and 2017. Private ignition data are restricted to fires that impacted USFS lands; fires that originated on private land and spread to other state or federal jurisdictions are not included.

**Figure 5:**
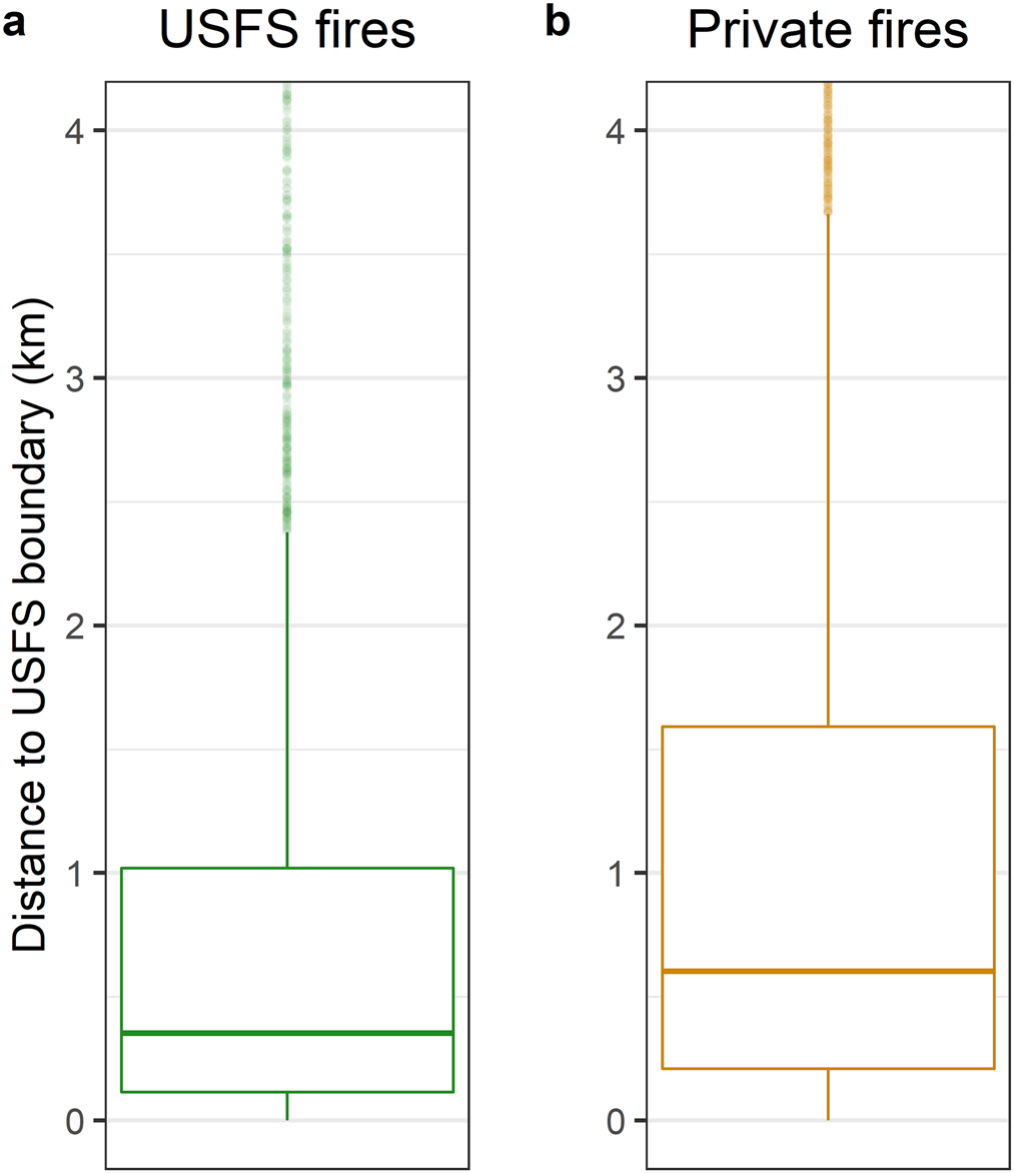
Distance from CB ignitions to USFS national forest borders for fires ignited on (**a**) USFS and (**b**) private lands between 1992 and 2017. To improve figure interpretability, the maximum distance shown here is constrained by the 90th percentile (2.62 km) of distance between a USFS ignition and national forest boundary. Private ignition data are restricted to fires that impacted USFS lands; fires that originated on private land and spread to other state or federal jurisdictions are not included.

Approximately 11% of all fires on national forest lands burned across USFS boundaries. Most CB ignitions were human-caused (e.g., debris burning, equipment use, escaped campfires) and originated on private lands (Table 1). CB ignitions were most abundant in parts of southern California where USFS lands abut dense population centers, and relatively rare in sparsely populated landscapes such as Wyoming and Nevada, and cool, wet environments such as northwest Washington (Fig. 4). We quantified a CB ignition zone based on the distance most (90%) CB ignitions occurred from a USFS boundary. The CB ignition zone extended 2.6 km within USFS lands and 4 km outside of USFS lands (Fig. 5).

**Table 1:** We identified a total of 22,026 CB fires that impacted USFS lands.

Ignition location	Lightning-caused		Human-caused		Total	
	# fires	% of total	# fires	% of total	# fires	% of total
Private	3036	14	10,235	46	13,271	60
USFS	2059	9	4052	18	6111	28
Other	997	5	1647	7	2644	12
Total	6092	28	15,934	72	22,026	100

The majority (88%) originated on either USFS or private lands, and the remainder started on other ownerships (e.g., state, city, other federal). Most CB fires were caused by humans (e.g., debris burning, equipment use, escaped campfires) on private lands.

**Figure 6:**
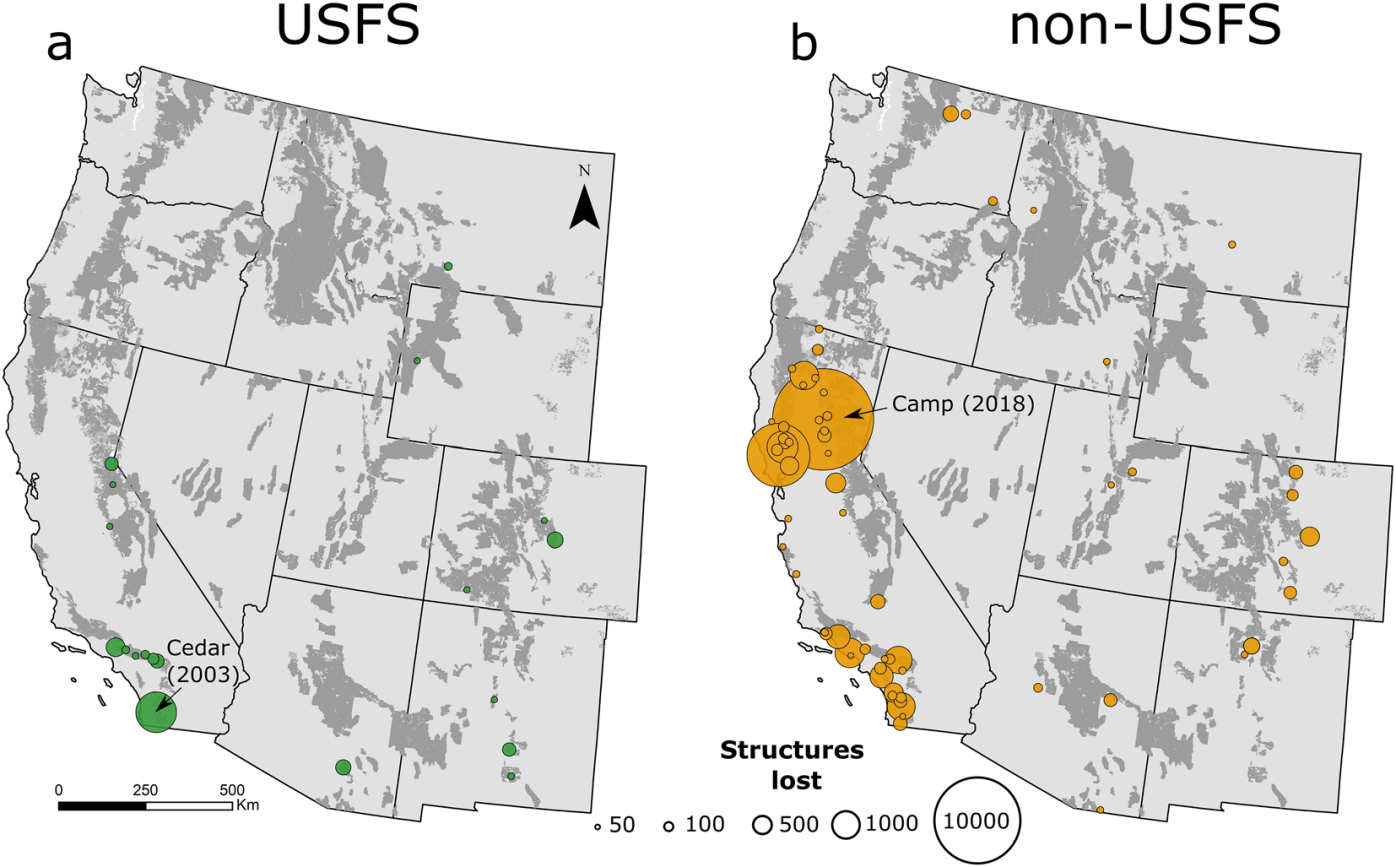
Location of destructive wildfires (> 50 structures lost) between 2000 and 2018 that originated on (**a**) USFS lands, and (**b**) non-USFS lands. Fire locations are symbolized by magnitude of structure loss. Relatively few destructive fires originated on USFS lands. The most destructive USFS and non-USFS fires during this time are the Cedar fire and the Camp fire, respectively.

A systematic inventory of fire-induced structure loss from ICS-209 and ancillary spatial datasets resulted in a list of 91 fires that destroyed more than 50 buildings between 2000 and 2018 (Fig. 6). Fires starting on USFS lands represent (24%) of destructive fires, and these fires were responsible for 14.7% (5077) of the total structures destroyed (34,493). Only two destructive fires ignited on USFS lands were caused by lightning, the remainder were started by humans, including energy infrastructure. The majority (63) of destructive fires occurred in California, most of which were human-caused on private lands.

**Figure 7:**
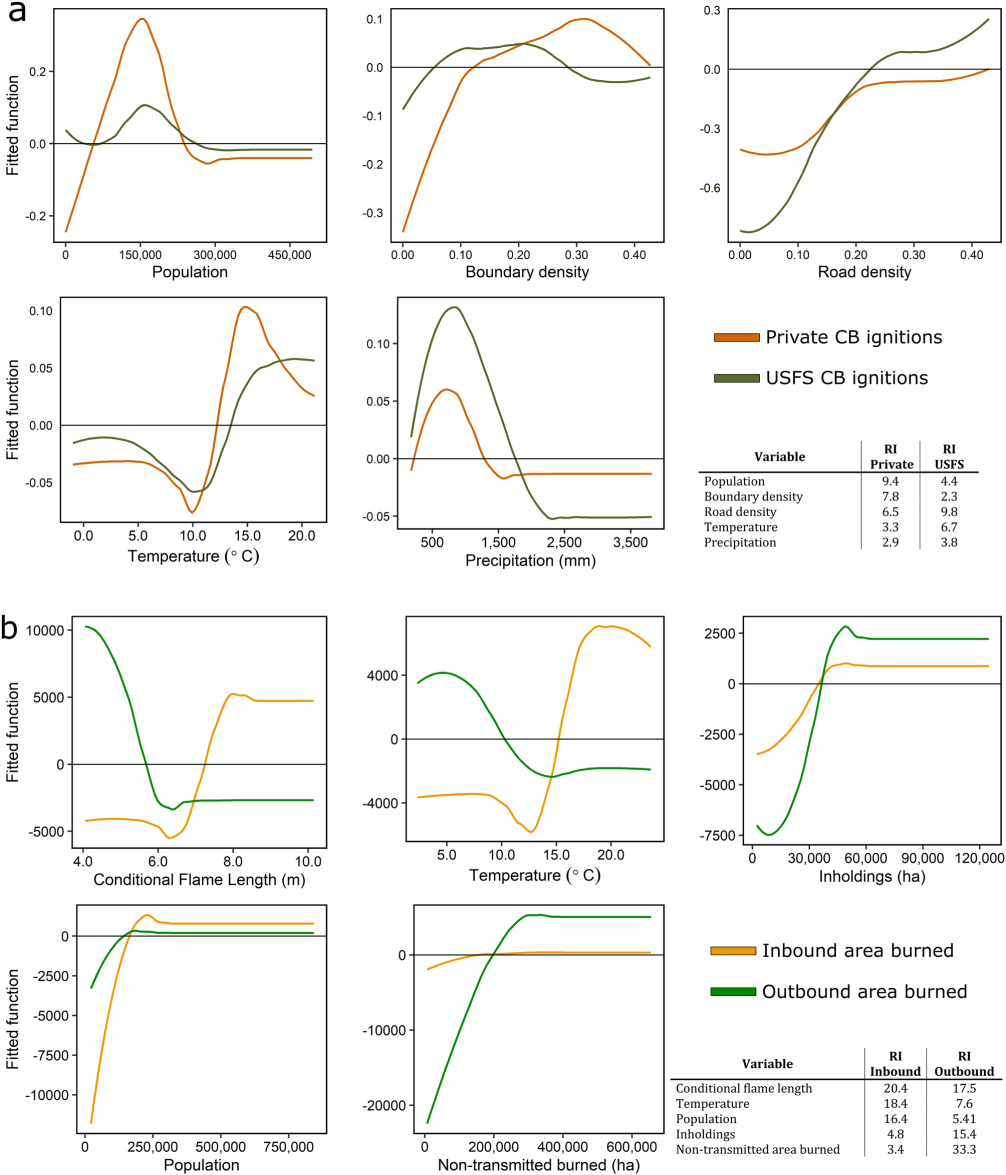
Partial dependence plots and relative influence of variables used to model (**a**) CB ignitions and (**b**) CB area burned. Note that the scales vary on the *y* axes, which represent each variable’s effect on (**a**) ignition counts and (**b**) CB area burned after accounting for the influence of other predictor variables. Predictions were center-scaled by subtracting the mean from each value. Partial dependence plots are shown in descending order of importance (left to right) determined by averaging variable relative importance (RI) values between models. Uninfluential variables (RI < 2.5) are not shown.

Boosted regression tree statistical modeling, a machine learning algorithm, demonstrated strong associations between CB fire activity and multiple social and biophysical factors (Table 2, Fig. 7). Models were fit for four response variables: (1) private CB ignitions, (2) USFS CB ignitions, (3) area burned by outbound CB fire, and (4) area burned by inbound CB fire. The performance of CB ignition models was very good (USFS deviance explained = 75%, cross-validated = 72%; private deviance explained = 84%, cross-validated = 75%). For the private ignitions model, population was the most important variable. Predicted ignitions displayed a hump-shaped response peaking at around 150,000 people within the CB ignition zone. Private ignitions increased sharply with jurisdictional boundary density and plateaued at moderate values. We observed a strong positive association between private ignitions and road density, and a weak positive association with average temperature. For the USFS ignitions model, road density was the most important variable and demonstrated a strong positive association with ignitions. Predicted USFS ignitions peaked in hot, dry locations with moderate population levels. There was evidence of residual spatial autocorrelation in both ignition models that was resolved when a residual autocovariate was added.

**Table 2:** Predictor variables for boosted regression tree (BRT) analyses.

Variable	Description	Source
Population^a^	Population within each sample area, averaged from 1990, 2000, and 2010 datasets	Radeloff et al.^4^
Road density	Data were rasterized; “road” cells were summed and divided by the area of the sample	https://www.here.com/
Boundary density^a^	Data were rasterized; “boundary” cells were summed and divided by the area of the sample	https://wfdss.usgs.gov/
Conditional flame length	Most likely flame length (m) at a given location if a fire occurs, based on wildfire simulations. Averaged across each sample area	Scott et al.^34^
Precipitation	Average annual precipitation (mm, 1981–2010) averaged across each sample area	PRISM
Temperature	Average daily mean temperature (°C, 1981–2010) averaged across each sample area	PRISM
Inholdings^b^	Area of non-USFS lands within national forest boundaries (ha) derived from jurisdictional spatial data	https://wfdss.usgs.gov/
Non-transmitted area burned^b^	Area burned (ha) by fires that did not spread beyond national forest borders to other ownerships	FIRESTAT

^a^For CB area burned models, these variables were only sampled in the 4-km external buffers around national forests.

^b^Variables only included in CB area burned models.

At the national forest scale, CB area burned model performance was comparatively worse (outbound deviance explained = 44%, cross-validated = 42%; inbound deviance explained = 32%, cross-validated = 30%), but we did observe important correlations. For the outbound model, the most important variable was area burned by non-transmitted (NT) fire, which was positively associated with outbound area burned up to intermediate values (~ 300,000 ha). A similar, but weaker relationship was observed between outbound area burned and inholdings. Fire intensity, represented by conditional flame length, was negatively associated with outbound area burned up to six meters, beyond which the response leveled off. Similarly, outbound CB area burned peaked at around 7 °C. CB area burned was relatively low at average annual temperatures greater than 15 °C. For the inbound area burned model, conditional flame length was the most important variable; CB area burned increased sharply as flame lengths increased from six to eight meters. Inbound area burned increased substantially between 12 and 18 °C average annual temperature. Population exhibited a strong positive association with inbound area burned up to approximately 300,000 people. The relationship between inbound area burned and inholdings was similar to the outbound model. Evidence of residual spatial autocorrelation in the inbound model was addressed with the addition a residual autocovariate. The outbound model did not exhibit residual spatial autocorrelation.

## Discussion

Our study provides the first region-wide empirical assessment of CB fire transmission patterns in the western US. By leveraging multiple fire databases, we were able to identify ownership at ignition for CB fires that burned both USFS lands and other ownerships. The magnitude and directionality of CB fire transmission varied substantially across our study area, but overall, CB fires were more likely to originate on private lands than USFS lands. CB ignitions, area burned, and structure loss were all concentrated in parts of California, where approximately two-thirds of CB fire activity occurred on USFS lands from fires originating on other ownerships. Our findings do not support the assertion that a majority of the most destructive fires spread from USFS-managed wildlands to communities. Broad-scale statistical modeling of CB ignitions and area burned provided evidence that human development patterns are strongly associated with CB fire activity. The population of the CB ignition zone surrounding national forests in our study area increased by 39% between 1990 and 2010, and our results indicate that CB fire risk will likely continue to increase as human development expands into sparsely populated landscapes^4^. Our findings highlight the need for increased cross-scale multiparty risk governance and CB pre-fire planning to minimize the social and economic damages of CB fire while maintaining ecologically beneficial burning^37,38^.

Our analysis provides an important empirical compliment to simulation studies based on large numbers of hypothetical fire events. Consistent with our results, modeling studies report that CB fire risk to communities is highest in parts of California^23,39,40^, and that community/private land tenures, rather than public lands, contribute the most fire risk to structures in the western US^24,41^. In contrast, our results differ in some important ways from simulation modeling studies. For instance, Palaiologou et al. 2019 reported high rates of simulated USFS to private fire transmission around the perimeters of the Gila, Apache-Sitgreaves, Tonto, Prescott, Coconino, and Kaibab national forests in the southwestern US^24^. Our analysis does not provide empirical evidence of substantial USFS to private fire transmission in these areas. A similar study reported that over 500 structures were exposed (but not necessarily lost) to fires spreading from these southwestern national forests *each year*^23^. However, our structure loss analysis only identified two fires that resulted in > 50 structures lost that involved these national forests between 2000 and 2018 (Yarnell Hill, 2013; Rodeo-Chediski, 2002). In their most recent comparison of empirical versus simulated wildfire impacts in the West, Ager et al. (2021)^39^ found good alignment in annual area burned estimates, while building exposure was substantially overestimated in simulations^37^. Of course, asking what did happen and what might happen are different endeavors. The relevant empirical record for the former is necessarily limited to occurrences within the past few decades, while wildfire simulation systems provide realizations of tens of thousands of hypothetical contemporary fire-seasons, including some far more extreme than observed. Moreover, the nuances of fire protection efforts specific to the WUI and associated communities are not well captured in simulation systems, but likely help lessen actual exposure^39^. Clearly both empirical and simulated data are important for assessing CB fire risk. When combined, empirical and simulation analyses can contribute to holistic representations of where socio-ecological CB fire linkages have emerged in the past, and where they may be likely to develop or be reinforced in the future.

CB fire risk transmission is strongly mediated by human development patterns as a function of human-caused ignitions, road and boundary networks, and the distribution of high-value assets potentially exposed to CB fire^23–25,41^. In our analysis, CB ignitions peaked at intermediate population values, and CB area burned was low in very sparsely populated landscapes. These findings are consistent with observations that fire occurrence increases with population up to a threshold beyond which fire activity declines—a phenomenon attributed to frequent human-caused ignitions in moderately populated areas and reduced fire activity associated with highly fragmented fuels and abundant fire suppression resources in densely populated areas^16,34^. Likewise, CB ignitions increased with jurisdictional boundary density up to a certain point, but declined where boundaries were densest, which again may be attributable to the decreased potential for fire spread in discontinuous fuels and greater suppression effort around extensive human development. National forests with abundant inholdings experienced more CB fire than those with few inholdings, indicating that smaller land tenures like inholdings are not only more likely to receive incoming fire^24^, they also appear to be sources of fire transmission to national forest lands. CB ignitions and area burned increased with road density, which we attribute to increased human-caused ignitions along road corridors that provide easy access to flammable vegetation in and around national forests^42^. The USFS has increasingly resorted to restricting access to entire national forests and even entire national forest regions to reduce the likelihood of human-caused ignitions during periods of high fire danger^43^. Decommissioning or limiting public access to roads may be another approach to limiting human-caused ignitions, but successful implementation would require input from fire managers who frequently utilize roads for access, fuel breaks, and pre-fire operational planning^44^, as well as buy-in from the public who use roads for recreation access and other purposes.

Biophysical gradients were strongly associated with CB fire activity after accounting for social factors. CB ignitions were more common in hotter and drier climates, with some indication that CB fire occurrence was limited by fuel availability in the least productive, hot, dry locations^45^. Non-transmitted fire activity was a strong predictor for outbound area burned, but only weakly associated with inbound area burned, indicating that national forest fire load may not be an appropriate metric for prioritizing CB fire risk mitigations designed to protect valued natural resources on public lands. We were surprised to find that outbound and inbound area burned were associated with substantially different biophysical contexts. Outbound fire activity increased at simulated low fire intensities (i.e., light, flashy fuels) in hot, dry environments, while inbound fire increased at simulated high fire intensities (i.e., tall brush and timber fuels) in cooler, moister contexts. Both of these fire behavior environments present challenges to fire managers (e.g., high rates of spread; intense, long duration burning), but it is not clear why they would differentially influence fire transmission^46^. It is likely that our broadscale analysis of CB area burned was partly confounded by variability at spatial scales smaller than those measured^47^. Presently, comprehensive USFS CB fire activity data are not available at smaller spatial scales, and additional research is needed to determine the influence of a more comprehensive suite of biophysical factors on CB area burned at finer resolutions.

An empirical understanding of the geography of CB fire activity can provide a common operating picture for multiparty risk management oriented around which actors can most efficiently reduce aspects of risk^48^. One of the wildfire risk reduction strategies commonly proposed to prevent fires from spreading from federally-managed wildlands to communities is the reduction of hazardous fuels^49,50^. In some contexts, strategically placed fuel treatments can reduce fire severity and local fire spread^51,52^. However, fuel treatments are not ecologically appropriate in many fire-prone ecosystems and their effectiveness at landscape scales is limited^2,31^. Wildfires rarely interact with treatments before fuels recover to hazardous levels^53–55^, and treatments are generally not designed to be effective during the extreme weather and fire behavior conditions associated with the small number of large, destructive fires that escape initial containment^31^. Federal agencies can also influence management on state and private lands through recently established collaborative authorities and strategic frameworks (e.g., Wyden Act, National Cohesive Strategy, Good Neighbor Authority, Shared Stewardship), but federal land managers are poorly positioned to incentivize community fire adaptation relative to state and local actors^56,57^.

“The USFS manages over 67 million hectares interspersed among other land tenures across the western US, necessitating the agency’s engagement in CB wildfire risk management. However, the USFS could benefit from a critical evaluation of where it can meaningfully direct its resources to mitigate risks within the context of its mission, span of control, and authority. Rather than direct a majority of resources to the structure loss problem, which can be fundamentally decoupled from the land management problem^31,58^, the USFS could instead emphasize forest health, resilience, and the natural amenity values that sustain communities and livelihoods. Given that (1) most CB ignitions are caused by humans on private lands, (2) high structure loss fires ignited on USFS lands are relatively rare, and (3) fire-induced structure loss is increasing despite substantial suppression and fuel reduction expenditures^31,59^, CB fire risk to communities in particular may be best defined in terms of minimizing potential damages to developed high value assets like homes, and best oriented towards private lands, homeowners, and communities^31,58^. Prevention, hazardous fuel treatments, and suppression will remain important components of CB fire risk management strategies in many landscapes. However, based on the near ubiquity of fire transmission in fire-prone landscapes, escalating suppression expenditures, WUI expansion, and positive feedbacks between human development and CB fire risk, eliminating CB fire transmission is probably not operationally feasible and may not be ecologically desirable or socially effective31. There are a multitude of high-value assets on federally managed wildlands, such as water supplies, critical habitat, recreation infrastructure, and other natural and cultural resources, which may be better protected or enhanced through the reintroduction of fire rather than continued emphasis on control^2^”.

Land managers and communities may be best served by adapting to increasing CB wildfire in the western US, rather than attempting to minimize fire transmission^2,38^. Based on the empirical evidence presented here, reducing exposure and increasing the resilience of fire-adapted communities with the assumption that wildfire is inevitable seems like a more realistic approach than attempting to exclude fire based on the mistaken assumption that more fire suppression expenditures will result in less fire activity^30,31^. Community wildfire protection plans (CWPPs) are one of the key mechanisms designed to accomplish the National Cohesive Strategy’s goal of human populations and infrastructure that can withstand wildfire without loss of life and property^27,60^. Nearly all CWPPs focus on fuels reduction and the creation of fire breaks designed to prevent wildfire from spreading from wildlands to communities, instead of efforts to reduce the ignitability of homes and other values at risk when fires do spread to populated areas. In contrast to these CWPP implementations, our findings and others’ suggest that private landowners and communities are best positioned to develop and maintain communities that can withstand wildfire by minimizing the likelihood of home ignition, preventing human-caused fires from occurring in the WUI, and limiting development in high risk areas through land use planning and zoning regulations ^31,58,61,62^**.**

While communities and private land owners appear to occupy the nexus of CB fire risk management, the social and ecological linkages created by CB fire necessitate engagement from all stakeholders at multiple scales^25^. Effective CB fire risk management strategies will likely require different strategies tailored to specific multijurisdictional contexts and based on localized systemic analyses, including, but not limited to, fuel treatments on public lands, exposure reduction on private lands, and the prevention and suppression of human-caused ignitions in high-risk locations on all ownerships. This shift in risk management emphasis may call for a reexamination of the most appropriate role for the USFS in some areas, perhaps to a mode of leveraging wildfire risk science to better frame problems and convening dialogues around co-management of wildfire risk.

Recent advances in wildfire risk science used in conjunction with empirical assessments of CB fire activity can help align risk management with the wildfire reality in multijurisdictional landscapes. When combined with local experiential knowledge, spatially explicit decision support tools like quantitative risk assessments^63^, suppression difficulty maps^64^, and potential control location atlases provide stakeholders with a common operating picture that can be used as the basis for co-managing risk^30,65^. Mapping the components of fire risk allows all landowners to simultaneously assess their exposure to wildfire and their contribution to the exposure of adjoining jurisdictions^66,67^. Transparency in pre-fire planning and risk governance can help build consensus around which actors are responsible for managing specific components of CB fire risk. Additionally, a shared CB fire risk knowledge base can be leveraged across state, county, and local scales to develop land use planning and zoning regulations designed to prevent development in areas where wildfire risk is unacceptably high^30,68^.

That much of the research on CB fire has focused on community exposure^23,25,69^ stems from the inherent imbalance between the values at risk in communities and the values at risk in publicly managed wildlands. Often it is considered more important to protect homes than it is to protect wildland ecosystems^31^. Rebuilding communities after wildfire is a challenging, complex process that can take years^70^. Meanwhile, post-fire ecological recovery can take decades^71^, or never occur at all if pre-fire vegetation can’t reestablish due to repeat burning, unfavorable climate, or a lack of surviving seed sources^72,73^. Our analysis was not designed to evaluate the ecological impacts of CB fire activity, and future empirical work on CB wildfire risk may therefore wish to consider the impacts of CB fire on a broader set of ecosystem processes and functions.

Future work could also add clarity on the prevention and response dimensions of CB fire risk. For instance, are closures and restrictions on non-USFS lands viable and would they measurably affect patterns of human-caused ignitions? Recognizing that fire response on mixed ownership landscapes entails a patchwork of entities, factors not explored here relate to the authorities, objectives, capacities, and capabilities of various response organizations (see Artley 2009)^26^. It may be the case that the USFS can effectively manage risk of outbound transmission due to factors like more robust planning and information systems and dedicated fire staff. Mapping a topography of the response system, how these and other factors vary, and how response to federal versus non-federal ignitions vary could be illuminating in this regard.

Wildfire and its controls are non-stationary, and the utility of past trends for forecasting future CB fire activity is probably limited^74^. In some ways, simulation modeling studies share this limitation because these models are parameterized with historical fuels, weather, and ignitions data^40^. While not taking historical patterns as givens, we anticipate fire transmission will continue to increase given directional trends in climate, the number of human-caused large fires, and human development near national forest boundaries^7,17^. Structure loss is also increasing^59^, but this trend may not be inevitable if the focus of wildfire governance can be shifted away from fire exclusion and towards reducing the likelihood of losses when fires invariably occur^31,58^.

## Conclusion

Our empirical assessment of CB fire activity can support the development of strategies designed to foster fire-adapted communities, successful wildfire response, and ecologically resilient landscapes. Adapting to increasing CB wildfire in the western US will require viewing socio-ecological risk linkages between CB fire sources and recipients as management assets rather than liabilities. We believe that a shared understanding of CB fire dynamics, based on empirical data, can strengthen the social component of these linkages and promote effective governance. The current wildfire management system is highly fragmented^74^, and increased social and ecological alignment between actors at multiple scales is necessary for effective wildfire risk governance^14,30^. Cross-boundary fire activity can contribute to multijurisdictional alignment when fire transmission incentivizes actors to collaboratively manage components of risk that manifest outside their respective ownerships^15^. A broader acknowledgement that CB is inevitable in some fire-prone landscapes will ideally shift the focus away from excluding fire in multijurisdictional settings towards improved cross-jurisdictional pre-fire planning and reducing the vulnerability of high-value assets in and around wildlands^30,31^. Federal agencies like the USFS can provide capacity, analytics, and funding, but given that private lands are where most high-value assets are located and where most CB fires originate, communities and private landowners may be best positioned to reduce losses from CB wildfire.

## Methods

### Study area

We analyzed CB fire transmission to and from 74 national forests in 11 western US states. Lands managed by the USFS not designated as national forests were excluded (e.g., Lake Tahoe Basin Management Unit). National forest lands in the western US are part of a diverse mosaic of land tenures consisting of private, state, and tribal and other federal ownerships. National forest lands in our study area cover 57 million ha and contain a wide variety of forest and rangeland ecosystems spanning broad climatic and fire regime gradients.

### Data sources

#### Cross-boundary wildfires

We identified CB fires using data from the Fire Statistics System (FIRESTAT). The FIRESTAT database contains a record for every fire with which the USFS was involved. FIRESTAT area burned data are classified into three coarse ownership categories: USFS lands, non-USFS lands not protected by the USFS, and non-USFS lands for which the USFS has protection responsibility pursuant to inter-agency protection exchange agreements. FIRESTAT records have spatial location information for the reported points of origin for most fires, but those data vary in format and precision (i.e., ranging from only Public Land Survey System subsection attributions to GPS-based latitude and longitudes). It also includes NFS unit (i.e., forest) codes and names that have changed over time. We therefore leveraged standardization and quality control procedures used to produce the national Fire Program Analysis Fire-Occurrence Database (FPA FOD), which is a compilation of wildfire records from local, state, and federal fire reporting systems, including FIRESTAT (Short 2017)^75^. At the time of our analysis, data were only available for fires that occurred between 1992 and 2017. FPA FOD procedures and data were used to attribute spatial ignition and nominal NFS unit data to CB fires. We restricted our FIRESTAT analysis to fires that occurred between 1992 and 2019 to align with the start year of the FPA FOD timespan, which is 1992 due to concerns about completeness and quality of spatial data prior to that year^76^.

#### Incident Status Summaries

Incident Command System Incident Status Summary Forms (ICS-209) report daily fire and suppression resource characteristics for significant wildfires. We used ICS-209 reports to identify the most destructive incidents between 2000 and 2018 using a threshold of 50 or more structures lost. We assigned a total structure loss count to each wildfire as well as the jurisdiction of the point of origin to evaluate whether or not the fire originated on USFS lands. A small number of destructive fires originated from multiple ignition sources located in different jurisdictions. When needed, we consulted local fire managers to properly attribute ownership for these event (see supplementary material). Where possible, we augmented ICS-209 destroyed structure counts with spatial building loss data. See Caggiano et al. (2020) for details^56^.

#### Statistical modeling data

We modeled CB fire activity in relation to predictor variables representing climate, fire intensity, human development, and jurisdictional boundary patterns. Average annual temperature and annual precipitation (1981–2010) were acquired from PRISM^77^. We used conditional flame length data derived from simulation modeling to represent average potential fire behavior^36^. Road density data was calculated based on HERE roads shapefile data (https://www.here.com). Jurisdictional boundary density data was calculated based on the Wildland Fire Decision Support System boundary shapefile data (WFDSS, https://wfdss.usgs.gov). We rasterized the road and boundary datasets and divided the number of “presence” cells (i.e., road, or jurisdictional boundary) by the total number of cells in each sample unit to generate density values. We calculated the abundance of inholdings using WFDSS jurisdictional boundary spatial data, and we derived area burned by non-transmitted fire from FIRESTAT data. To quantify population, we averaged 1990, 2000, and 2010 population estimates from wildland urban interface data developed by Radeloff et al.^4^.

### Analysis

#### Quantifying fire transmission

CB fires were identified as fires that burned both USFS lands and other ownerships based on FIRESTAT data. We aggregated FIRESTAT area burned data by the three ownership categories described above (USFS, non-USFS, non-USFS protected) to quantify the magnitude of CB fire transmission for each of the national forests in our study area and for our study area as a whole. Additionally, we summarized area burned by ownership for each year between 1992 and 2019 to evaluate temporal trends in CB fire transmission.

#### Mapping fire transmission

We assessed the geographic distribution of fire transmission by mapping national forests in our study area in terms of inbound and outbound area burned. Additionally, we mapped CB ignitions that originated on either USFS or private lands. These two ownership categories were the dominant sources and recipients of CB fire in our study domain. To attribute CB ignition ownership as precisely as possible, we linked CB fires identified from FIRESTAT to FPA FOD spatial fire origin data based on a shared, unique identifier. FPA FOD ignition location data were used to extract more detailed ownership information from the Protected Areas Database of the United States^78^ and 2019 Census Block Groups data (Wildland Decisions Support System, https://wfdss.usgs.gov). We assessed the geographic distribution of CB fire ignitions by summarizing the density of ignitions by ownership within a 20-km resolution hexagonal tessellated grid. Lastly, we leveraged the combined FPA FOD-FIRESTAT CB ignition spatial data to determine the distance between both private CB ignitions and USFS CB ignitions and the closest national forest boundary. We used the 90th percentiles of these two datasets to delineate a “cross-boundary ignition zone,” which we then utilized as the spatial extent for sampling predictor variables used in the statistical analyses described below.

#### Attributing ownership to destructive fires

We used FPA FOD data, state fire agency documentation, and news articles to attribute ownership to destructive fires. Where possible, we also assigned a cause to each destructive fire (e.g., lightning, arson). In some cases, it was very difficult to determine a specific ownership category for fires that did not ignite on federal lands (e.g., private, state, county, or city lands). This did not pose a significant problem because our primary objective was to differentiate between fires ignited on or off USFS lands. Attributing ownership to fire complexes (multiple fires managed as one incident) also presented a challenge because complexes were sometimes composed of fires that were ignited on different jurisdictions. Two complexes we are aware of (Okanogan Complex, BTU Lightning Complex) consisted of fires that originated on both USFS and non-federal lands. In both cases, we classified these fires as “non-USFS” based on available data and conversations with local fire managers. The USFS ignitions in these complexes either constituted a small minority of all ignitions (BTU Lightning Complex) or did not substantially impact communities (Okanogan Complex). Removing these fires, or changing their ownership classification, would not substantially alter our results or our interpretation.

#### Statistical modeling

We analyzed CB ignitions and CB area burned in relation to a suite of social and biophysical predictor variables representing climate, fire intensity, human development, and jurisdictional boundary patterns (Table 2). We fit models for the following four response variables: (1) private CB ignitions, (2) USFS CB ignitions, (3) area burned by outbound CB fire, and (4) area burned by inbound CB fire. CB ignitions were modeled at the scale of the 20-km resolution hexagonal grid used for mapping ignitions. Private and USFS CB ignition counts derived from the FPA FOD database were summed within every grid cell intersecting a national forest boundary. We normalized ignition counts by hexagon area where grid cells were clipped by the extent of our study area. We modeled CB fire activity at the national forest scale using inbound and outbound (protected and non-protected combined) area burned data from FIRESTAT. CB area burned predictor variables were sampled within the CB ignition zone identified using FPA FOD spatial data and described above.

We used boosted regression trees (BRT) to assess the relative importance of predictor variables and relationships between predictor variables and our four CB fire response variables. Models were fit for the appropriate family for each data distribution (ignition counts: Poisson, area burned: Gaussian), and parameterized to ensure at least 1000 trees were produced during the fitting process (learning rate = 0.001, tree complexity = 5, bag fraction = 0.5). We assessed model performance based on modeled and tenfold cross-validated percentages of deviance explained, which indicates the goodness of fit between modeled values and observed values^79^. We evaluated the importance of predictor variables using relative influence values and we used partial dependence plots to interpret the effects of predictor variables on the response after accounting for the average effects of all other variables in the model^80^. We tested for residual spatial autocorrelation using Moran’s I, and models with evidence of residual spatial autocorrelation were fit with a residual spatial autocovariate^79^. We assessed collinearity between predictors using a correlation matrix (see supplementary material). We considered variables to be highly correlated beyond a threshold of r >|0.7|, the point at which collinearity begins to meaningfully distort BRT model outputs^81^. In several cases we observed strong correlations between predictor values sampled inside and outside of national forest boundaries. To avoid possible model distortion and to simplify model interpretation, we averaged interior and exterior precipitation, temperature, and conditional flame length values prior to modeling. We conducted all analyses in R (ver. 4.0.3, R Core Team 2019). BRT modeling was performed using gbm^82^ and dismo^83^ R packages.

## Supplementary Information

Supplementary Information.
