# Supplementary material for: Human ignitions on private lands drive USFS cross-boundary wildfire transmission and community impacts in the western US
Source: Sci Rep. 2022 Feb 15;12:2624. doi: 10.1038/s41598-022-06002-3 (PMC8847424; doi:10.1038/s41598-022-06002-3)
Supplement: Supplementary file 1 — Supplementary Information. [file 41598_2022_6002_MOESM1_ESM.docx]

**Supplemental material for:**

**Human ignitions on private lands drive USFS cross-boundary wildfire transmission and community impacts in the western US**

William M. Downing*, Christopher J. Dunn, Matthew P. Thompson, Michael D. Caggiano, Karen C. Short

*Corresponding to: willmdowning@gmail.com

Submitted for review: Nature Scientific Reports

**Contents**

**Figure S1**. Correlation matrix for predictor variables included in CB ignition models

**Figures S2**. Correlation matrix of predictor variables included in CB area burned models

**Table S1**. Description of ownership attribution methods and fire name, state, ignition ownership, year, number of structures lost, and cause for fires that destroyed more than 50 structures in the western US between 2000 and 2018.


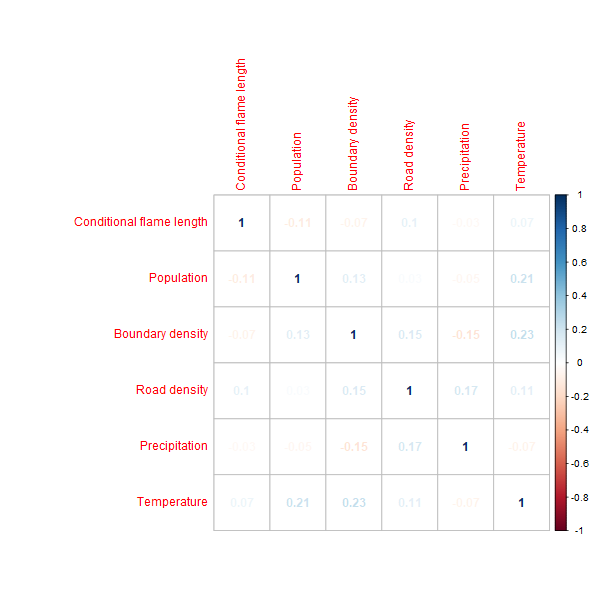


**Figure S1**. Correlation matrix (Pearson’s r) for predictor variables included in CB ignition models


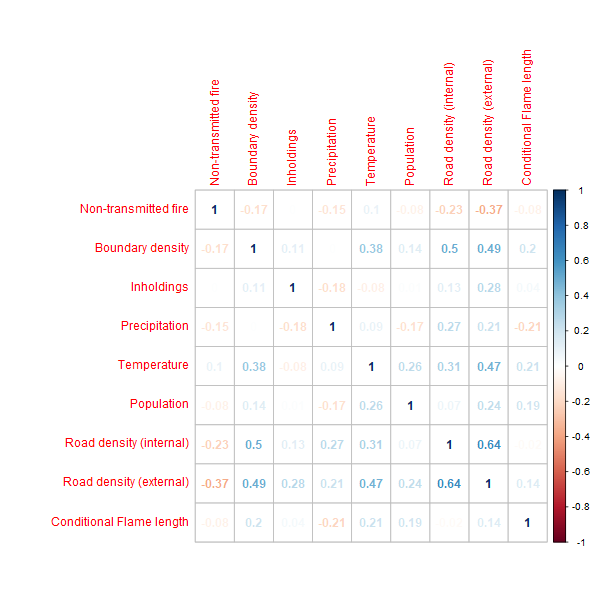


**Figure S2**. Correlation matrix (Pearson’s r) of predictor variables included in CB area burned models.

**Attributing ownership to destructive fires:** We used FOD data, ICS-209, structure loss spatial data, state fire agency documentation, and news articles to attribute ownership, cause, and structures lost to destructive fires. These data were gathered from sources of varying quality, and they are not appropriate for any analyses not described in the paper. In some cases, it was very difficult to determine a specific ownership category for fires that did not ignite on federal lands (e.g., private, state, county, or city lands). This did not pose a significant problem because our primary objective was to differentiate between fires ignited on or off USFS lands. All fires that were not started on federal lands were categorized as “STATE/PRIVATE.” Attributing ownership to fire complexes (multiple fires managed as one incident) also presented a challenge because complexes were sometimes composed of fires that were ignited on different jurisdictions. We are aware of only two destructive fires in our dataset that consisted of ignitions originating on both USFS and non-USFS lands. Based on discussions with local fire managers, we determined it was appropriate to classify both of these complexes as non-USFS fires, either because only one fire in the complex originated on USFS lands (BTU Lightning Complex), or because the fires that originated on USFS lands were not responsible for substantial structure loss (Okanogan Complex).**Table S1**. Fire name, state, ignition ownership, year, number of structures lost, and cause for fires that destroyed more than 50 structures in the western US between 2000 and 2018.

| **Fire name** | **State** | **Ignition ownership** | **Year** | **Structures lost** | **Cause** |
| --- | --- | --- | --- | --- | --- |
| Cerro Grande | NM | NPS | 2000 | 235 | Human |
| High Meadow | CO | USFS | 2000 | 51 | Human |
| Scott Able | NM | USFS | 2000 | 64 | Human |
| Iron Mountain | CO | STATE/PRIVATE | 2002 | 100 | Human |
| Missionary Ridge | CO | USFS | 2002 | 57 | Human |
| Rodeo | AZ | BIA | 2002 | 225 | Human |
| Williams | CA | USFS | 2002 | 62 | Human |
| Aspen | AZ | USFS | 2003 | 322 | Lightning |
| Cedar Fire | CA | USFS | 2003 | 2232 | Human |
| Grand Prix | CA | STATE/PRIVATE | 2003 | 135 | Human |
| Old Fire | CA | STATE/PRIVATE | 2003 | 993 | Human |
| Padua | CA | STATE/PRIVATE | 2003 | 65 | Human |
| Paradise | CA | OTHER FEDERAL | 2003 | 221 | Human |
| Bear | CA | STATE/PRIVATE | 2004 | 80 | Human |
| School | OR | STATE/PRIVATE | 2005 | 109 | Human |
| Derby | MT | USFS | 2006 | 86 | Lightning |
| Angora | CA | USFS | 2007 | 254 | Human |
| Corral | CA | NPS | 2007 | 53 | Human |
| Grass Valley | CA | USFS | 2007 | 178 | Human |
| Harris | CA | STATE/PRIVATE | 2007 | 253 | Human |
| Poomacha | CA | BIA | 2007 | 147 | Human |
| Rice | CA | STATE/PRIVATE | 2007 | 500 | Human |
| Santiago | CA | STATE/PRIVATE | 2007 | 725 | Human |
| Slide | CA | USFS | 2007 | 272 | Lightning |
| Witch | CA | STATE/PRIVATE | 2007 | 1061 | Human |
| BTU Lightning Complex | CA | STATE/PRIVATE | 2008 | 106 | Lightning |
| Freeway Complex | CA | STATE/PRIVATE | 2008 | 190 | Human |
| Humboldt | CA | STATE/PRIVATE | 2008 | 87 | Human |
| Sayre | CA | USFS | 2008 | 487 | Human |
| Summit | CA | STATE/PRIVATE | 2008 | 63 | Human |
| Tea | CA | STATE/PRIVATE | 2008 | 210 | Human |
| Trigo | NM | USFS | 2008 | 59 | Human |
| 49 | CA | STATE/PRIVATE | 2009 | 50 | Human |
| Jesusita | CA | STATE/PRIVATE | 2009 | 80 | Human |
| Station | CA | USFS | 2009 | 89 | Human |
| Fourmile Canyon | CO | STATE/PRIVATE | 2010 | 167 | Human |
| Las Conchas | NM | STATE/PRIVATE | 2011 | 63 | Human |
| Monument | AZ | NPS | 2011 | 62 | UKN |
| Charlotte | ID | STATE/PRIVATE | 2012 | 66 | Human |
| Dahl | MT | STATE/PRIVATE | 2012 | 73 | Lightning |
| High Park | CO | STATE/PRIVATE | 2012 | 259 | Lightning |
| Little Bear | NM | USFS | 2012 | 242 | Lightning |
| Ponderosa | CA | STATE/PRIVATE | 2012 | 69 | Lightning |
| Taylor Bridge | WA | STATE/PRIVATE | 2012 | 70 | Human |
| Waldo Canyon | CO | USFS | 2012 | 347 | Human |
| Wood Hollow | UT | STATE/PRIVATE | 2012 | 56 | Human |
| Black Forest | CO | STATE/PRIVATE | 2013 | 509 | Human |
| Clover | CA | BLM | 2013 | 68 | Human |
| Yarnell Hill | AZ | BLM | 2013 | 114 | Lightning |
| Boles | CA | STATE/PRIVATE | 2014 | 157 | Human |
| Carlton Complex | WA | STATE/PRIVATE | 2014 | 322 | Lightning |
| Courtney | CA | USFS | 2014 | 61 | Human |
| Butte | CA | STATE/PRIVATE | 2015 | 545 | Human |
| Clearwater Complex | ID | STATE/PRIVATE | 2015 | 50 | Lightning |
| Okanogan Complex | WA | STATE/PRIVATE | 2015 | 123 | Lightning |
| Rocky | CA | STATE/PRIVATE | 2015 | 97 | Human |
| Valley | CA | STATE/PRIVATE | 2015 | 1280 | Human |
| Blue Cut | CA | USFS | 2016 | 105 | Human |
| Chimney | CA | STATE/PRIVATE | 2016 | 71 | Human |
| Clayton | CA | STATE/PRIVATE | 2016 | 188 | Human |
| Erskine | CA | STATE/PRIVATE | 2016 | 285 | Human |
| Soberanes | CA | STATE/PRIVATE | 2016 | 57 | Human |
| Central LNU Complex | CA | STATE/PRIVATE | 2017 | 5303 | Lightning |
| Creek | CA | STATE/PRIVATE | 2017 | 146 | Human |
| Detwiler | CA | STATE/PRIVATE | 2017 | 63 | Human |
| Helena | CA | BLM | 2017 | 80 | Human |
| Lilac | CA | STATE/PRIVATE | 2017 | 114 | Human |
| Neu Wind Complex | CA | STATE/PRIVATE | 2017 | 238 | Human |
| Redwood | CA | STATE/PRIVATE | 2017 | 50 | Human |
| Southern LNU Complex | CA | STATE/PRIVATE | 2017 | 463 | Lightning |
| Sulfur | CA | STATE/PRIVATE | 2017 | 150 | Human |
| Wall | CA | STATE/PRIVATE | 2017 | 95 | Human |
| Camp | CA | STATE/PRIVATE | 2018 | 13696 | Human |
| Carr | CA | STATE/PRIVATE | 2018 | 1080 | Human |
| Dollar Ridge | UT | STATE/PRIVATE | 2018 | 90 | Human |
| Donnell | CA | USFS | 2018 | 54 | Human |
| Klamathon | CA | STATE/PRIVATE | 2018 | 85 | Human |
| Ranch | CA | STATE/PRIVATE | 2018 | 155 | Human |
| Roosevelt | WY | USFS | 2018 | 55 | Human |
| Spring Creek | CO | STATE/PRIVATE | 2018 | 216 | Human |
| Thomas | CA | STATE/PRIVATE | 2018 | 775 | Human |
| West | CA | STATE/PRIVATE | 2018 | 52 | Human |
| Woolsey | CA | STATE/PRIVATE | 2018 | 1187 | Human |
| Kincade | CA | STATE/PRIVATE | 2019 | 175 | Human |
| Sandalwood | CA | STATE/PRIVATE | 2019 | 76 | Human |
